# Having breakfast has no clinically relevant effect on bioelectrical impedance measurements in healthy adults

**DOI:** 10.1186/s12937-023-00882-5

**Published:** 2023-10-31

**Authors:** Julia W. Korzilius, Sosha E. Oppenheimer, Nicole M. de Roos, Geert J. A. Wanten, Heidi Zweers

**Affiliations:** 1 Department of Gastroenterology and Hepatology – Dietetics, Radboud University Medical Center, Geert Grooteplein Zuid 10, Nijmegen, 6525 GA The Netherlands; 2 Division of Human Nutrition and Health, Wageningen University, Droevendaalsesteeg 4, Wageningen, 6708 PB The Netherlands; 3 BIA Workgroup of Nutritional Assessment Platform, Amsterdam, the Netherlands

**Keywords:** Anthropometry, Body composition, Bioelectrical impedance analysis, Fat-free mass, Fasting

## Abstract

**Background:**

Bioelectrical impedance analysis (BIA) is commonly used to evaluate body composition as part of nutritional assessment. Current guidelines recommend performing BIA measurements in a fasting state of at least 2 h in a clinical setting and 8 h in a research setting. However, since asking patients with malnutrition or sarcopenia to fast is not desirable and literature to support the strategy in the guidelines is lacking, this study aimed to assess the impact of breakfast on BIA measurements.

**Methods:**

We performed an explorative, prospective study in healthy volunteers aged between 18 and 70 years, with a normal fluid balance and a body mass index between 18.5 and 30 kg/m^2^. BIA measurements were performed according to the standard operating procedure in the fasting state, and 1, 2, 3, and 4 h after ingesting a standardized breakfast meal of about 400 kcal with a 150 mL drink, using the hand-to-food single-frequency BIA (Bodystat500 ®). The Kyle formula was used to calculate the primary outcome, i.e. fat-free mass (FFM, kg). A linear mixed model was used to compare baseline values with other time points. A difference of 1 kg in FFM was considered clinically relevant.

**Results:**

Thirty-nine (85% female) volunteers were included, with a median age of 28 years (IQR 24–38). In 90% of the participants, having breakfast had no clinically relevant impact on the estimated FFM. For the group, the most pronounced mean difference, a statistically but not clinically significant higher value of 0.2 kg (0.4%), was observed after 3 h of fasting compared to baseline. No statistically significant differences were found at the other time points.

**Conclusion:**

Eating affects single-frequency BIA measurements, but differences in FFM remain below clinical relevance for most participants when using a standardized breakfast. Thus, the current study suggests performing a BIA measurement in a fasting state is not required.

**Supplementary Information:**

The online version contains supplementary material available at 10.1186/s12937-023-00882-5.

## Background

Several methods are available to estimate body composition, including dual-energy X-ray absorptiometry (DXA), computed tomography (CT), air displacement plethysmography (ADP), and bioelectrical impedance analysis (BIA). BIA is a non-invasive and relatively cheap method that can easily be applied in clinical settings due to portability of the required device [1, 2]. BIA measures resistance and reactance; these variables are used in a formula to estimate a person’s fat-free mass (FFM) and fat mass (FM) [3]. The principle of BIA is based on the difference in resistance between tissues; fat and bone yield higher resistance, while water-containing tissues like blood and muscle have a higher conductivity [2].

Although BIA is not the most reliable method to measure body composition when compared to DXA, CT, or ADP, it is the easiest to apply in a clinical setting. Standardizing the different factors that influence the measurement is key to improve precision, e.g., placing of electrodes, body position, and electrolyte abnormalities can influence the measurement [4]. Hence, a Standard Operating Procedure (SOP) is used for standardization [5]. The SOP and European Society for Clinical Nutrition and Metabolism (ESPEN) guidelines state that the measurement should be performed after a fasting period of at least 8 h in a research setting and 2 h in clinical settings [5, 6]. BIA is frequently applied in patients with malnutrition or sarcopenia; however, measuring in a fasted state is undesirable in this situation [7, 8].

The recommendation of fasting before BIA measurements in the ESPEN guideline (2004) is based on three studies [6]. Deurenberg et al. (1988) evaluated the effect of a liquid-formula meal on BIA and reported a mean impedance decrease of 13–17 Ω, representing a 3.3% change [9]. Additionally, Fogelholm et al. (1993) found an increase of 0.6% in resistance 1-h post-meal, followed by a significant decrease of about 5 and 4 Ω at 2.5 and 4 h post-meal, respectively [10]. Kushner et al. (1996) concluded that, depending on the experimental condition, impedance might decrease 4–15 Ω over 2–4 h after a meal [11]. Another study, not mentioned in the guideline by Gallagher et al. (1988), concluded that fasting is necessary due to a decrease in impedance after consumption of a breakfast meal [12]. Thus, the evidence for 8 h of fasting mentioned explicitly in the guideline is scarce. Moreover, all studies use impedance or resistance as the primary outcome, which is not directly clinically relevant.

Given the limited literature on the necessity and timing of fasting when performing BIA measurements and the urge to prevent fasting in patients with malnutrition or sarcopenia, we aimed to assess whether fasting leads to clinically relevant differences in FFM estimation when performing single-frequency (SF) BIA measurements.

## Methods

### Study design and participants

This exploratory, observational, multi-centre non-inferiority study in healthy participants was conducted from September to December 2022. The study was designed to determine the necessity and timing of fasting before a BIA measurement. Participants were recruited through advertisement and word of mouth within the Gastroenterology and Hepatology – Dietetics department at Radboud university medical center (Radboudumc) and the Department of Dietetics at HAN University of Applied Sciences. People were eligible to participate if they were aged between 18 and 70, with a body mass index (BMI) of 18.5–30 kg/m^2^. Due to possible interference with the BIA measurement, people were excluded if they were breastfeeding or pregnant, had a pacemaker or defibrillator, used medication influencing fluid balance, had an abnormal fluid balance, or suffered from burn wounds or decubitus. All subjects provided their written informed consent before participation.

### Study procedures

**Fig. 1:**
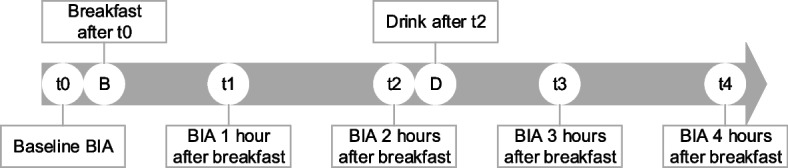
Study timeline

During the study, participants underwent BIA measurements at five different time points. The initial measurement (t0) was performed after an overnight fast of at least 8 h and after determining the participant’s height (InLabS50, InBody, Seoul, South Korea). Before each measurement, participants were questioned about their adherence to the study protocol. After this, the participant received their chosen standardized breakfast. This was either full-fat yoghurt (200 g) with granola (50 g) and raisins (15 g) (388 kcal) or two slices of brown bread (70 g) with 30 + matured cheese (63 g) and margarine (10 g) (400 kcal). Remaining measurements were performed 1, 2, 3 and 4 h after breakfast (t1, t2, t3 and t4, respectively). Participants drank coffee, tea or water during breakfast and after t2 (150 ml). During the remaining part of the study, participants could not eat or drink anything. Figure 1 shows an overview of the study timeline.

### Bioelectrical impedance analysis measurement and calculations

BIA measurements were conducted following the SOP for SF-BIA using a hand-to-foot Bodystat® 500 (Bodystat, UK) [5]. Before each BIA measurement, participants were asked to urinate, after which their weight (kg) was measured with one layer of clothing and without shoes on a Seca® 877 scale. A correction of 1 kg for clothes was applied. At each time point, the participant was measured three times consecutively to determine the variation within time points. We used the mean of the three measurements to calculate the additional variables. FFM and FM were calculated using the Kyle formula [3]. Afterwards, both FFM and FM were divided by squared height to obtain the index (kg/m^2^): FFM index (FFMI) and FM index (FMI).

### Outcomes

Primary outcome was the difference in FFM between the baseline (t0) and the other time points (t1, t2, t3 and t4). Secondary outcomes were the differences between baseline (t0) and the other time points (t1, t2, t3, t4) for FM, FFMI, FMI, weight, impedance, reactance, resistance, and phase angle. A difference of ≥ 1 kg in FFM and FM was considered clinically relevant, as determined by BIA experts of the Dutch BIA workgroup Nutritional Assessment Platform [3, 4].

### Statistical methods

No sample size calculation was performed because we were not so interested in detecting (minor) mean group differences but rather sought to focus on the individual effects of having breakfast as well as the proportion of individuals with acceptable differences in that regard in the setting of a pilot study. According to the literature, including thirty participants was sufficient to perform a pilot study [13]. Continuous variables were presented as mean with standard deviation (SD) or median and interquartile range (IQR) in case of not-normal distribution. Binary variables were described as percentages.

For primary and secondary outcomes, linear mixed models were applied to test whether baseline measurements differed from the other time points. Participant ID was the random variable, and time point was the fixed variable, with t0 as a reference. Sidak adjustment was applied to correct for multiple testing. The residuals were checked for normality with histograms. Bland–Altman plots were generated, showing the mean difference and limits of agreement (LOA).

Furthermore, independent t-testing was performed to determine whether there was a difference between the types of breakfast regarding the change in FFM estimation. The coefficient of variation (CV, %) for FFM was calculated within and between time points. A p-value < 0.05 was considered statistically significant. Statistical analyses were performed using IBM SPSS Statistics for Windows, version 27 (IBM® Corp., Armonk, NY, USA).

### Ethical approval

This study was reviewed by the research ethics committee of the Radboudumc in Nijmegen, the Netherlands (reference number 2022–15782). The committee declared this study was not subject to the Medical Research Involving Human Subject Act. Reporting was according to STROBE guidelines [14].

## Results

### Demographics

In total, 39 adults between 19 and 66 years participated. Most participants had a healthy BMI ranging from 18.7 to 29.7 kg/m^2^. Baseline characteristics of the participants are presented in Table 1. Thirty-one participants chose the yoghurt breakfast (80%), while eight chose the bread breakfast (21%).

**Table 1:** Baseline characteristics of participants

Participant characteristics	***n* = 39**
Female – no. (%)	33 (85)
Age – years, median (IQR)	28 (24–38)
Weight – kg, mean (SD)	67 (9.6)
Height – m, mean (SD)	1.74 (0.07)
BMI – kg/m^2^, median (IQR)	21.9 (20.1–24.1)

*Abbreviations*: *BMI* Body mass index, *IQR* Interquartile range, *No* Number, *SD* Standard deviation

### Fat-free mass

**Fig. 2:**
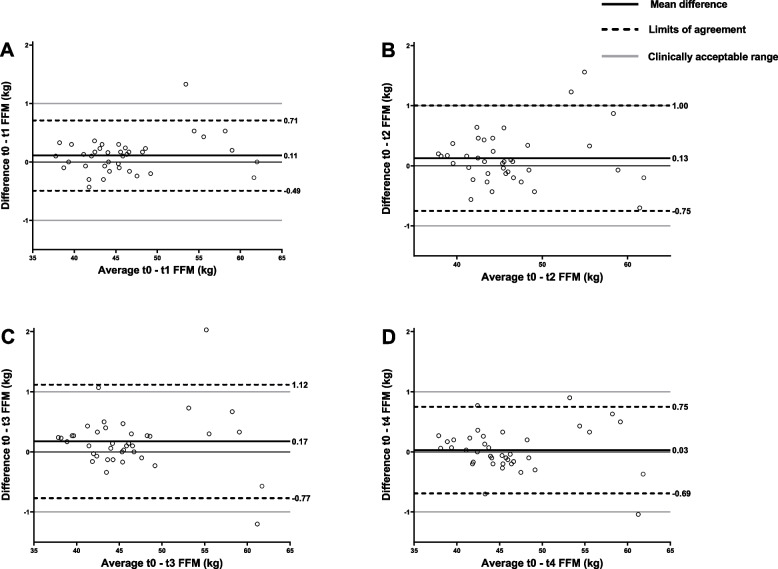
Bland–Altman plots showing the difference in fat-free mass between the baseline measurements (t0) and t1 (**A**), t2 (**B**), t3 (**C**), and t4 (**D**). Lines represent the mean difference, the limits of agreement and clinically acceptable range

No statistically significant difference was found 1 and 2 h after breakfast compared to baseline (Table 2). FFM estimation increased by 0.4% (0.2 kg) after 3 h (Fig. 2C). The mean FFM estimation returned to baseline values after 4 h (Table 2). For all four time points, the difference between fasted and non-fasted FFM measurements was not clinically relevant, with a difference of < 1 kg of FFM in 90% of participants. The CV was 4.7 times higher between time points (CV = 0.42%) than the average CV of the three measurements within one time point (CV = 0.09%).

**Table 2:** Means (SD) of all outcomes at baseline (t0) and 1 to 4 h after breakfast ingestion (t1-4)

Outcome	**Baseline (t0)**	**t1**	**t2**	**t3**	**t4**
Weight – kg, mean (SD)	67.3 (9.6)	67.5 (9.6)*	67.3 (9.6)	67.3 (9.6)	67.1 (9.6)*
FFM – kg, mean (SD)	46.2 (6.2)	46.3 (6.3)	46.3 (6.3)	46.4 (6.2)*	46.2 (6.2)
FM – kg, mean (SD)	21.1 (5.7)	21.2 (5.7)	21.0 (5.7)	20.9 (5.7)*	20.9 (5.7)*
FFMI – kg/m^2^, mean (SD)	15.23 (1.45)	15.26 (1.46)	15.27 (1.43)	15.29 (1.43)*	15.24 (1.44)
FMI – kg/m^2^, mean (SD)	6.98 (1.89)	7.01 (1.90)	6.96 (1.93)	6.93 (1.92)	6.92 (1.90)*
Impedance – Ω, mean (SD)	617 (61)	615 (62)	613 (60)	611 (59)*	614 (59)
Reactance – Ω, mean (SD)	63.3 (6.8)	63.2 (6.6)	63.1 (6.9)	62.9 (6.8)	63.1 (6.5)
Resistance – Ω, mean (SD)	614 (61)	612 (62)	610 (60)	608 (59)*	611 (59)
Phase angle – °, mean (SD)	5.9 (0.5)	5.9 (0.5)	5.9 (0.5)	5.9 (0.5)	5.9 (0.5)

*Abbreviations*: *SD* Standard deviation, *FFM* Fat free mass, *FM* Fat mass, *FFMI* Fat free mass index, *FMI* Fat mass index

^*^p < 0.05 in bold, compared to t0

### Secondary outcomes

Average FM estimates decreased significantly by 0.9% at 3 and 4 h after breakfast, resulting in a 0.2 kg reduction (Table 2, Additional file Fig. 1). No differences were found between breakfast meals for either outcome.

## Discussion

This study explored the effect of breakfast on FFM estimation and found no clinically relevant difference between fasted and non-fasted measurements. In 90% of participants, FFM estimates changed less than 1 kg compared to their fasting value. In four participants, the difference exceeded the pre-set limit of 1 kg, of whom two admitted to having breached the research protocol. The most pronounced mean difference in FFM was found after 3 h; this was statistically significant but not clinically relevant, remaining well below 1 kg difference. We hypothesized that this difference after 3 h was due to the uptake of the breakfast at this time. In the first 2 h after breakfast, most of the food remains in the stomach, which would not affect impedance outcomes [15]. However, body weight increased due to the breakfast mass, influencing the calculation since weight is a variable in the Kyle formula [3].

The results of our study are consistent with findings of Hollander-Kraaijeveld et al. (2020, *n* = 84), in cystic fibrosis patients. These authors found a mean decrease of 0.2 kg in FFM after eating a non-standardized meal, with a difference of < 1 kg of FM and FFM in 86% of the patients [16]. Although the trends compared to our study are in different directions in these (metabolically) substantially differing subject populations, the average change was minor and not clinically relevant. Furthermore, both studies found opposing individual responses, with participants increasing or decreasing FFM estimation after eating.

Androutsos et al. (2015, *n* = 55) studied the effect of a high-fat meal or high-carbohydrate meal on impedance and FM estimation [17]. These authors reported that average FM increased most after 2 h, increasing 0.8 kg with a median difference of 4.8%. While the present study found a decrease in FM estimation, both studies have found non-clinically relevant changes (Table 2).

Our results are consistent with the three studies mentioned in the ESPEN guideline [9–11]. These all found differences in resistance and impedance outcomes before and after eating. However, none of these differences appeared clinically relevant, i.e. leading to treatment changes. In hindsight, in our opinion, we never had robust evidence to let our patients fast. Thus, all studies in the literature report similar results, while concluding on statistically significant differences in BIA outcomes but without clinically relevant differences between fasted and non-fasted BIA measurements.

In our study, a difference of ≥ 1 kg in FFM was considered clinically relevant, whereas Hollander-Kraaijeveld et al. used ≥ 1.5 kg as a clinically relevant difference. If we also defined 1.5 kg as a clinically relevant difference, we would only have one outlier left, which empowers the argument of measuring in a non-fasting state.

According to the literature, there is a within-day variability of 1–2% of resistance when performing an SF-BIA measurement and a weekly intra-person variability of 2–3.5% [3]. Comparing these values with the CV of 0.42% in FFM between time points found in this study, the variation due to having breakfast is below the within-day and the weekly variability. This finding further supports our opinion that measuring in a fasted state is unnecessary.

Weight increased on average by 0.2 kg 1 h after breakfast and decreased by 0.2 kg after 4 h (Table 2). This increase was due to the weight of the breakfast, while the decrease can be explained by losing water due to urinating before each measurement. There was no statistically significant change in reactance, while resistance and impedance decreased by 6 Ω after 3 h (Table 2). This indicates that this change caused the increased FFM estimation at t3, rather than weight, since the average weight was similar at baseline and at this time point.

This study has limitations, including the homogeneity of our study population, mostly compromising of females aged 24–38 with a healthy BMI. This homogeneity reduces the external validity of this study, but there is no evidence to suggest that there would be other findings in other types of populations. Furthermore, participants could choose between two breakfast meals. Differences in composition between the meals could have affected the BIA measurements differently, although we found no difference between breakfast groups regarding all outcomes. Hollander-Kraaijeveld et al. also did not find clinically relevant differences, and their participants had no limitations regarding nutritional intake [16]. Finally, we cannot be completely certain whether all subjects correctly reported their adherence to the study protocol.

Concerning the strengths, the research was standardized, following the SOP. Moreover, all measurements were performed by the same researcher, and on each study day, the SF-BIA was calibrated using the same device throughout the study.

## Conclusion

In conclusion, these results indicate important implications for BIA measurements since, depending on the study protocol, it is feasible to include non-fasted subjects without negatively impacting study quality. Based on all the currently available literature and our data, we advise removing the advice on fasting from the current guideline. This facilitates body composition measurements in more patients, thereby enabling personalized patient care. Future studies should combine all these data to provide evidence as to whether the implemented nutritional treatment leads to improved patient outcomes, especially in the vulnerable population of patients suffering from malnutrition or sarcopenia.

### Supplementary Information

**Additional file 1:** **Figure 1. **Bland-Altman plots showing the difference in fat mass between the baseline measurements (t0) and t1 (A), t2 (B), t3 (C), and t4 (D). Lines represent the mean difference with the limits of agreement (LOA).

## Data Availability

The datasets used and/or analysed during the current study are available from the corresponding author on reasonable request.

## Abbreviations

DXA: Dual-energy X-ray absorptiometry
CT: Computed tomography
ADP: Air displacement plethysmography
BIA: Bioelectrical impedance analysis
FFM: Fat-free mass
FM: Fat mass
SOP: Standard Operating Procedure
ESPEN: European Society for Clinical Nutrition and Metabolism
SF: Single-frequency
BMI: Body mass index
FFMI: Fat-free mass index
FMI: Fat mass index
SD: Standard deviation
IQR: Interquartile range
LOA: Limits of agreement
CV: Coefficient of variation
