# Supplementary material for: Having breakfast has no clinically relevant effect on bioelectrical impedance measurements in healthy adults
Source: Nutr J. 2023 Oct 31;22:55. doi: 10.1186/s12937-023-00882-5 (PMC10617110; doi:10.1186/s12937-023-00882-5)
Supplement: Supplementary file 1 — Additional file 1: Figure 1. Bland-Altman plots showing the difference in fat mass between the baseline measurements (t0) and t1 (A), t2 (B), t3 (C), and t4 (D). Lines represent the mean difference with the limits of agreement (LOA). [file 12937_2023_882_MOESM1_ESM.docx]

**Additional file**

**Figure 1** – Bland-Altman plots showing the difference in fat mass between the baseline measurements (t0) and t1 (A), t2 (B), t3 (C), and t4 (D). Lines represent the mean difference with the limits of agreement (LOA).
